# Supplementary material for: Are carbon emissions trading and green financial instruments synergistic? -Comprehensive quantitative research based on content analysis
Source: PLoS One. 2024 Mar 7;19(3):e0298601. doi: 10.1371/journal.pone.0298601 (PMC10919601; doi:10.1371/journal.pone.0298601)
Supplement: S1 Data — (ZIP) [file pone.0298601.s001.zip › Data/地级市Z_F工作报告.zip!/╔╜╢1⁄2/╝├─╧╩╨/└┤╫╘╣1⁄2╓┌║┼í╛┬φ┐╦╩2╛▌═°í┐_2011.docx]

各位代表：

　　现在，我代表市人民政府向大会报告工作，请连同《济南市国民经济和社会发展第十二个五年规划纲要(草案)》一并审议，并请各位政协委员和其他列席人员提出意见。

　　一、“十一五”时期省会现代化建设取得新的成就

　　“十一五”时期的五年，是我市发展史上极不平凡的五年。在中共济南市委的坚强领导下，市政府与全市人民一起，以科学发展观为统领，按照“维护省城稳定、发展省会经济、建设美丽泉城”的总体思路，解放思想，提升境界，抢抓机遇，积极作为，在克服困难中推进省会经济社会发展不断取得新成就。2010年，全市生产总值达到3910.8亿元，比“十五”末翻一番，年均增长13.9%；地方财政一般预算收入266.1亿元，是“十五”末的2.5倍，年均增长20.2%；社会消费品零售总额达到1725.5亿元，是“十五”末的2.1倍，年均增长16.4%；全社会固定资产投资累计完成7226.6亿元，是“十五”时期的2.6倍，年均增长18%。全面完成“十一五”规划确定的目标任务。

　　(一)发展方式加快转变。认真贯彻落实国家和省应对国际金融危机的决策部署，制定实施了扩内需促增长、加快发展方式转变的一系列政策措施，全市经济持续平稳向好。产业结构发生积极变化，三次产业比例由“十五”末7.3∶45.9∶46.8调整为5.5∶41.9∶52.6。现代服务业占服务业比重达到44.6%，比“十五”末提高2.8个百分点，金融、信息、旅游产业年均增长超过20%，会展业发展加快。工业结构优化提升，形成交通装备、电子信息、机械装备3个过千亿元的主导产业，高新技术产业产值跨上2千亿元新台阶，占工业总产值的比重达到41.5%，比“十五”末提高11.4个百分点，新信息、新能源、生物医药、高端装备制造等战略性新兴产业加快发展。投资结构不断优化，着力增加交通、能源、农业、水利、社会民生、高新技术、现代服务业等领域投资。2010年，现代服务业和技术改造投资分别增长25%和47.8%；民间投资增长30.6%，占全部投资的比重提高5个百分点。创新驱动能力增强，深入开展国家知识产权工作示范城市、国家创新型城市、中国软件名城等试点工作，综合性国家高技术产业基地、国家创新药物孵化基地和国家综合性新药研发技术大平台建设加快推进。五年来，新增市级以上企业技术中心110家、工程技术中心133家、创新型企业104家，专利四项指标居全省首位。高新区各项经济指标大幅增长，在全国排名明显前移。节能减排成效明显，五年来，淘汰落后水泥产能240万吨，完成任务目标的148%；淘汰落后炼钢产能150万吨、落后炼铁产能83万吨，完成任务目标的100%；关停小火电40.2万千瓦，完成任务目标的104%；削减化学需氧量3.01万吨、二氧化硫4.44万吨，全面完成“十一五”节能减排目标。

　　(二)城市发展跨越提升。把推进城市规划建设管理作为扩内需、调结构、惠民生的重要抓手，抢抓筹办第十一届全国运动会和第七届国际园林花卉博览会的重大机遇，拓展发展空间，打造发展载体，提升了城市功能形象。五年来累计投入2000亿元，比“十五”时期翻一番。完成新一轮城市总体规划、土地利用和矿产资源总体规划修编，实现中心城区控规全覆盖，城乡规划体系基本形成。全面推进老城区改造提升，突出抓好棚户区、城中村和危旧简易楼改造。启动三大新区建设，东部新区奥体文博片区初具规模，西部新区西客站片区建设全面展开，滨河新区小清河综合整治工程扎实推进，16个城市综合体招商建设顺利，拉开了城市发展新框架。奥体中心“一场三馆”、园博园荣获中国建设工程最高奖。京沪高铁济南段铺轨完工，济菏高速、绕城大北环、北园快速路、二环东路高架、济阳和建邦黄河大桥等工程竣工。建成“两横三纵”快速公交网，城市公共交通年客运量达到8.64亿人次。五年来累计投入131亿元加强市政“四供两排”设施建设，是“十五”时期的2.9倍。升级改造67个供水低压片区管网，完成济西应急供水一期工程，新建及改建热源厂13座、污水处理厂7座。济南电网完成投资107.7亿元，供电能力达到450万千瓦，比“十五”末提高70%。大明湖扩建改造完成，护城河全线通航，趵突泉连续7年喷涌，泉城特色更加突出。启动国家森林城市创建活动，城市绿化覆盖率达37%。持续推进城市环境综合整治，实施城市绿化、美化、净化、亮化工程，开展城管“十大行动、百件实事”活动，以“八乱”治理为抓手，有效化解了一大批城市管理难点问题，完成108座破损山体治理，市区空气质量逐步改善。

　　(三)“三农”建设步伐加快。五年来，市级财政“三农”支出65.9亿元，是“十五”时期的3倍，加大了支农惠农政策力度。2010年粮食总产289.4万吨，连续8年丰产丰收。建设都市农业园区和特色品牌基地77个，规模以上农业龙头企业达到350家，农产品质量安全水平提升。新农村建设“十大行动”全面完成，基本实现村村通油(水泥)路、通客车、通有线电视和新农合全覆盖，自来水入村率超过95%，五保对象集中供养率达到75.8%。完成38个小城镇建设任务，启动农村整村改造迁建项目326个，新建农村住房16.4万户、改造危房2.6万户。实施农村扶贫开发项目210个，基本消除年纯收入1000元以下的绝对贫困人口。“百库除险”工程全面完成，治理水土流失4.2万公顷，发展节水灌溉1.266万公顷。防治美国白蛾、秸秆利用禁烧工作成效显著，重大动物疫病防控扎实有效。章丘连续6年跻身全国中小城市科学发展百强市，商河等县经济社会和城区建设发生新变化。

　　(四)改善民生成效显著。坚持把保障和改善民生作为根本出发点和落脚点，全市人民在改革发展中得到更多实惠。生活水平明显提高，2010年城市居民人均可支配收入和农民人均纯收入分别达到25321元和8903元，年均增长13.3%和13.1%。居民消费价格总水平上涨2.1%。采取积极政策促进就业，五年来，新增城镇就业67.8万人，农村富余劳动力转移就业88.8万人次，城镇登记失业率控制在4%以内。加快推进社会保障体系建设，市财政五年投入社保资金83.4亿元，是“十五”时期的2倍。企业退休养老金从“十五”末月均782元提高到1520元，城乡低保实现应保尽保，新型农村养老保险参保人数达到82万，农民工参加医疗、工伤保险人数达到37.2万和54万。启动38个集中片区和34个零星片区棚户区改造，开工建设安置房290万平方米，解决了6万户18.3万困难群众住房问题，一大批长期居住在低矮潮湿环境的群众生活条件得到改善。启动沁园新居等6个公共租赁住房项目，总建筑面积140万平方米，向7339户家庭发放廉租住房补贴6110万元。市级财政投入151亿元，连续3年完成为民办实事33件。

　　(五)社会事业全面加强。人才强市战略深入实施，推进“5150引才计划”，引进海内外高层次人才99人，16人进入“新世纪百千万人才工程”，高技能人才队伍建设得到加强。优化教育资源配置，推进幼儿园标准化建设和中小学校舍安全工程，努力解决外来务工子女入学难问题，对农村困难学生和涉农专业学生实行免学费政策，全市普及高中阶段教育。医疗卫生事业成效显著，认真落实国家基本药物制度，推进卫生监督体制改革，在全省率先实施“惠民医疗工程”，新农合政府补助标准达到每人每年120元。实施“卫生强基工程”，建成2366个标准化村卫生室和252所城市社区卫生服务中心(站)，有效防控甲型流感等重大疫情疫病。努力稳定低生育水平，人口自然增长率控制在2.78‰以内。繁荣发展文化事业和文化产业，启动“3810”工程，完成60个乡镇综合文化站及3400个农家书屋建设。成功举办第五届中国京剧艺术节和首届中国非物质文化遗产博览会，府学文庙修缮工程竣工，公共博物馆免费开放，创作推出《天下泉城》等一批优秀作品。圆满完成第十一届全国运动会济南主赛区各项筹办工作，充分展示了省会现代化建设成就和济南人民团结奋斗、争创一流的精神风貌。我市运动员在北京奥运会、广州亚运会、全运会等大型比赛中获金牌79枚，实现历史新突破。新闻出版、广播电视、社会科学、民族宗教、档案史志、妇女儿童、老龄、残疾人、地震、气象等工作取得新成绩。

　　(六)改革开放继续深化。扎实推进重点领域和关键环节改革，经济发展动力活力不断增强。市、县、镇(乡)三级机构改革顺利完成，审批制度改革不断深入。深化政府资金、行政事业资产管理和投融资体制改革，整合集中17项财政专项资金，98户市级行政事业单位资产统一运营管理，市公共资源交易平台建成运行。国有企业改革取得重要进展，非公经济比重达到42.6%。

　　粮食流通体制改革全面完成。开放型经济深入发展，累计实际到账外资37.5亿美元、进出口总额302.9亿美元，是“十五”时期的2.8倍和2.6倍，其中机电产品、高新技术产品出口占比分别达到61.8%和11.8%，比“十五”末提高27.3和8.3个百分点。累计完成对外承包和劳务合作营业额52.3亿美元。服务外包成为外向型经济发展新亮点，占全省比重40%以上。外事、侨务、对台工作取得新成绩，缔结友好合作城市关系17对，开通济南至大阪包机、台北直航，口岸设施功能不断完善。

　　(七)和谐社会创新发展。深入推进平安济南建设，重点加强社会矛盾化解、社会管理创新、公正廉洁执法，强化四级信访网络和维稳工作制度建设，认真协调处理群众信访问题。依法打击各类违法犯罪活动，加强交通消防安全管理，社会治安形势总体平稳，应急管理体系不断健全。深入开展专项整治活动，安全生产形势基本稳定。税收征管和纳税服务、产品质量和食品药品监管、工商管理服务都实现新的提升。广泛开展“迎全运、讲文明、树新风”等精神文明创建活动，涌现出泉城义工、贴心民政、阳光大姐等一批全国有影响的知名品牌和郑承镇、刘振华、刘延宝等先进道德模范，历下、市中、槐荫、章丘荣膺国家、省级和谐社区建设示范单位，我市在全国公共文明指数测评中位次大幅前移。国防教育和双拥共建成效显著，荣获双拥模范城六连冠。积极支持慈善事业发展。圆满完成擂鼓镇、北川县城灾后重建和对口援藏、援疆、帮扶重庆的各项任务。

　　(八)政府建设扎实推进。加快法治政府建设，坚持依法行政、科学民主决策，深入推进政务公开，积极推行重大决策公示、评估和听证制度，“五五”普法任务全面完成。自觉接受市人大及其常委会监督和政协民主监督，五年来办理人大代表建议1808件、政协委员提案2526件。加快服务型政府建设，提高行政效能，网上办事扎实推进，大力精简行政审批项目，市级行政审批由436项减少到93项，压减审批环节236个、办理时限1332个工作日，废止政府规章15件、规范性文件105件。建立行政执法电子监察系统，梳理规范了4326项处罚事项。推进采购代理机构竞争制度和电子化政府采购平台建设，五年来节约资金11.1亿元。连续四年面向市民和社会各界开展“我为《政府工作报告》建言献策”活动，开通12345市民服务热线，开展“下基层、解难题、办实事”、“执政为民、廉洁高效”和“创先争优”活动。注重从源头上预防和治理腐败，强化科技防腐，组建公共资源交易服务中心，为工程招投标、土地交易、产权交易、医药采购等提供统一的交易平台。加强监察审计监督，严格领导干部经济责任审计，节约资金11亿元。支持纪检监察和司法机关依法依纪查处腐败案件，五年来全市共处理机关工作人员319名，廉政建设不断加强。

　　各位代表！五年来的巨大变化，凝聚着全市人民的智慧和汗水。面对国际金融危机冲击和经济社会转型发展的复杂局面，我们同心同德，攻坚克难，不断取得新的成绩。这些成绩极大地增强了全市人民建设美好家园的自豪感和自信心，激励我们在建设现代化省会城市的道路上阔步前进。五年来的发展和进步，是中央和省委、省政府高度重视、亲切关怀的结果，是市委科学决策、正确领导的结果，是全市方方面面团结一致、共同努力的结果。在这里，我代表市政府，向给予政府工作大力支持的各级人大代表、政协委员，各民主党派、工商联、无党派人士和全体市民，向关心支持地方工作的人民解放军驻济部队、武警部队官兵和中央、省驻济单位、高校科研院所，向为济南发展作出贡献的港澳台同胞、海外侨胞、国内外朋友，向来济兴业的广大投资者和外来务工人员，表示崇高的敬意和衷心的感谢！

　　各位代表！五年来，全市经济社会繁荣发展，诸多领域取得了历史性突破，积累了弥足珍贵的经验。一是坚持深入贯彻落实科学发展观，着力转变发展理念，在保持经济平稳较快发展中不断提升城市整体素质。二是坚持解放思想，提升境界，着力创新思路，优化环境，改善服务，以思想境界大提升推动各项事业大发展。三是坚持以人为本、民生优先，全力为民办实事、办好事，让人民群众更好地分享改革发展成果。四是坚持城市建设与经济社会发展相结合，拓展发展空间、提升功能品位，为培育现代产业、发展社会事业、保障改善民生提供支撑条件。五是坚持用改革创新的办法解决发展中的问题，构建有利于科学发展的体制机制，为经济社会发展提供强大动力。这些深刻体会，需要在今后工作中继续坚持和发扬。

　　各位代表，在总结成绩的同时，也要清醒地看到，我们仍然面临许多制约科学发展的深层次矛盾和问题。主要是：经济综合实力不够强，发展质量效益还不高；经济结构性矛盾比较突出，城乡区域发展仍不协调；资源环境制约加剧，节能减排和生态环境建设面临更多考验；社会建设任务繁重，公共服务水平需要提高，在收入分配、社会保障、教育、医疗、住房等方面有许多问题亟待解决；各级领导干部驾驭科学发展的能力有待增强，思想境界有待进一步提升，政府自身建设、廉政建设还需大力加强。我们要高度重视这些问题，努力加以解决。

　　二、关于《济南市国民经济和社会发展第十二个五年规划纲要(草案)》的说明

　　“十二五”时期是全面建设小康社会的关键时期，是深化改革开放、加快转变经济发展方式的攻坚阶段，是我市实现跨越发展的重要战略机遇期。面对新的形势和任务，我们必须进一步解放思想、提升境界，精心谋划发展思路，主动应对环境变化，奋发有为，科学发展，力争经过5年努力，使我市经济结构更加优化，创新优势更加突出，城市功能更加完善，生态环境更加优美，社会发展更加和谐，努力打造实力济南、魅力济南、宜居济南，基本建成与山东经济文化强省相适应的现代化省会城市。

　　中共济南市委九届九次会议通过了《关于制定济南市国民经济和社会发展第十二个五年规划的建议》，提出了未来五年国民经济社会发展的奋斗目标、指导方针和主要任务。根据《建议》的精神，市政府制定了《济南市国民经济和社会发展第十二个五年规划纲要(草案)》，已提请大会审查。下面，我就几个问题作简要说明。

　　(一)“十二五”规划《纲要(草案)》编制过程和特点

　　市政府于2009年12月正式启动规划编制工作，组织有关部门、科研机构和各方面专家，对“十二五”时期经济社会发展的重大问题开展专题研究，对规划进行咨询论证。面向社会开展为“十二五”规划建言献策活动，人大代表、政协委员及社会各界提出了许多建议。《纲要(草案)》的编制过程，是发扬民主、集思广益、科学决策的过程。《纲要(草案)》力求反映经济社会发展的新形势、新特点和新要求，体现战略性和政策性，并明确政府的工作重点和责任。在指标设置上，强化科学性、导向性和约束性，调整完善了转方式、调结构的指标体系，增加了资源环境、民生改善等方面约束性指标。在规划实施上，突出内生增长和要素保障，强化重大项目支撑，注重健全完善规划实施机制。

　　(二)“十二五”时期经济社会发展的指导思想和主要目标

　　在全面总结“十一五”时期发展成果、客观分析国际国内形势基础上，《纲要(草案)》明确提出以科学发展为主题，牢牢把握加快转变经济发展方式这一主线，紧紧围绕“拓展城市发展空间、打造现代产业体系”的工作思路，加快推进经济转型、城市转型、社会转型，大力实施新型城市化、新型工业化、创新驱动和富民惠民战略，着力实现优化经济结构、加强社会建设、提升城市品位、保护生态环境、深化改革开放的新突破，全面建设更高水平的小康社会。《纲要(草案)》提出了“十二五”时期经济社会发展的主要目标，这些目标既与全面建成小康社会的目标相衔接，又反映了发展的阶段性特征和要求。

　　一是关于经济增长速度问题。《纲要(草案)》提出，今后五年生产总值年均增长11%。这个目标比“十一五”低，主要是综合考虑各方面因素，根据需要和可能提出的。“十二五”期间要始终保持经济平稳较快发展，把发展作为解决一切问题的前提和基础，实施扩大内需方针，加快形成消费、投资、出口协调拉动经济增长新格局。既要保持经济平稳较快发展，又要为加快经济发展方式转变和经济结构调整留有空间，把发展建立在经济结构优化、质量效益提升、资源消耗下降的基础上，正确处理速度和质量、效益的关系。这个目标是积极的，经过努力是可以实现的。

　　二是关于加快经济发展方式转变问题。针对资源环境压力日益加大和提高自主创新能力的需要，《纲要(草案)》增加了二氧化碳排放量、万元工业增加值用水量、氮氧化物排放量、耕地保有量、基本农田保护面积等约束性指标。这些指标体现了建设资源节约型、环境友好型社会的要求，是全面协调可持续发展的需要，是现实和长远利益的需要，必须保证完成。

　　三是关于改善民生问题。“十二五”期间人民生活水平提高要与经济发展同步，把保障和改善民生作为根本出发点和落脚点，加快推进基本公共服务均等化。《纲要(草案)》提出，城市居民人均可支配收入和农民人均纯收入年均增长11%，15岁以上人口平均受教育年限提高到12年，千人医疗机构床位数达到5.8床等，这些指标事关广大人民群众切身利益，是转方式、扩内需的重要内容，我们必须以更大的决心和力度取得新的进展。

　　(三)“十二五”时期的战略重点和主要任务

　　《纲要(草案)》对“十二五”时期全市经济、社会、生态、文化建设和改革开放等方面作出全面部署，提出了明确的任务目标和政策措施。

　　一是加快推进新型城市化。“十二五”时期，我市将进入城市化快速发展的新阶段。《纲要(草案)》提出，城市化率达到75%，坚持走集约化城市发展道路，把增创经济社会发展空间、促进区域协调发展、形成主体功能区作为“十二五”工作重点。做大做强中心城市，加快规模膨胀和功能提升，推进老城区和东部新区、西部新区、滨河新区规划建设，构建“一城三区”城市发展格局。坚持以工促农、以城带乡、城乡统筹，优化城镇发展格局，积极推进北跨发展，壮大县域经济，实施统筹城乡发展七大工程，提升新农村建设水平。发挥省会辐射带动作用，打造省会城市群经济圈。

　　二是打造现代产业体系。《纲要(草案)》提出了“54321”的发展思路，三次产业比例调整到4∶38∶58。把服务业打造成省会首位经济，是省委、省政府提出的工作要求，也是我市转方式调结构必须抓好的关键环节。构建五大区域性服务业中心是服务业加快发展的战略任务，培育四大战略性新兴产业是抢占经济科技制高点的重要举措，我们有基础、有优势、有潜力，必须抓住机遇，有所作为。

　　三是建设国家创新型城市。2010年我市被确定为国家创新型城市试点，担负着在全国先行先试的重要任务。《纲要(草案)》对此提出了明确目标和具体部署，主要是完善区域创新体系，建设国家综合性高技术产业基地，加强创新平台、园区建设，启动一批重大科技专项，培育一批优势产业集群，加强人才队伍建设。

　　四是进一步深化改革开放。《纲要(草案)》根据“十二五”时期面临的新形势，对推进重点领域和关键环节改革作出了全面部署，努力形成有利于转变经济发展方式、促进全面协调可持续发展的体制机制。按照统筹发展和对外开放的要求，实施更加积极主动的开放战略，扩大对内对外开放，构建开放型经济体系。

　　五是建设生态文明城市。面对日益严重的资源环境问题，必须加快构建资源节约、环境友好的生产方式和消费模式，增强可持续发展能力。《纲要(草案)》提出了明确的任务和措施，规划了五个生态功能区、创建国家森林城市“十大工程”和济南市水系生态环境区域布局，制定了加强节能降耗、推进资源节约及削减污染物排放等约束性指标，强调要制定实施低碳经济发展规划。我们必须以高度的紧迫感责任感做好这些工作，努力打造水清、天蓝、树绿、气爽的生态文明城市。

　　六是促进社会和谐稳定。改善民生是科学发展观的根本要求。“十二五”时期必须加大力度，在改善民生和构建和谐社会方面取得更大进展。《纲要(草案)》提出加快构建民生改善八大体系，推进城乡、区域、群体间基本公共服务均等化，加强公共安全建设，加强社会主义民主政治建设和文化建设，完善社会管理体制。经过五年努力，使我们的社会更加和谐，让人民群众生活得更加富裕美好！

　　三、2011年经济社会发展的主要任务

　　2011年是中国共产党成立九十周年，也是“十二五”时期开局之年，更是加快转变经济发展方式、保持经济平稳较快发展的关键一年。从宏观经济形势看，国际金融危机深层次影响依然存在，不稳定不确定因素仍然较多，改革进入攻坚时期，开放面临新的情况，稳增长、调结构、抗通胀、保民生压力很大。对我市来说，城市化和新型工业化加快推进，产业转型升级正孕育着新的突破，经济社会已进入转型发展的关键时期。我们既面临难得的历史机遇，也面临诸多可以预见和难以预见的风险。我们要清醒把握形势，积极应对各种困难和挑战，奋发有为地推进省会改革开放和现代化建设。

　　2011年全市经济社会发展的总体要求是：深入贯彻落实科学发展观，突出科学发展这一主题，把握加快转变经济发展方式这条主线，按照“拓展城市发展空间，打造现代产业体系”的工作思路，实施新型城市化、新型工业化、创新驱动、富民惠民四大战略，着力调结构、稳物价、保民生、促改革，保持经济平稳较快发展和社会和谐稳定。

　　2011年经济社会发展主要预期目标是：生产总值增长12%，地方财政一般预算收入增长15%，固定资产投资增长18%，社会消费品零售总额增长16%。服务业增加值增长13.5%，规模以上工业增加值增长12%，高新技术产业产值比重提高2个百分点。外贸出口增长12%，实际到账外资增长10%。城市居民人均可支配收入、农民人均纯收入均增长12%，居民消费价格总水平涨幅控制在4%左右，城镇登记失业率控制在4%以内，人口自然增长率控制在5‰以内。完成省政府下达的年度节能减排目标任务。

　　(一)保持经济平稳健康运行。完善消费增长机制，保持投资合理适度增长，增强经济发展的协调性和内生动力。一是增强居民消费能力。提高政府改善民生和社会事业支出比重，增加对城镇低收入群体和农民的补贴，适时提高最低工资标准，适当增加职工工资收入。继续实施家电下乡、以旧换新政策，做好建材下乡试点工作。培育消费热点，鼓励扩大新能源汽车等节能环保型产品消费，发展旅游、健身、教育、家政、文化娱乐等服务型消费业态。营造便利、安全、放心的消费环境，深入开展“满意消费惠万家”和诚信商贸企业创建活动，改造提升一批特色商业街，构建5至8分钟便民消费服务圈。推进农产品进城、万村千乡市场工程和大型连锁超市与农产品质量安全示范区对接。二是提高投资质量和效益。做好“十二五”前期投资项目衔接工作，抓好投资结构调整，把信贷资金更多投向实体经济特别是“三农”和中小企业。发挥重大项目对转方式调结构的支撑引领作用，加快推进一批重点项目建设，积极争取财政资金、信贷资金支持，鼓励扩大民间投资。严格执行用地、节能、环保等准入标准，严禁投资产能过剩行业。三是保持物价基本稳定。适当增加重要农副产品储备，稳定主要农产品、基本生活必需品和重要生产资料的生产和供应，强化农产品产运销衔接，提高粮食、食用油应急保障能力。建立完善消费品生产、流通、消费信息监测、预警和发布制度，严肃查处恶意炒作、哄抬价格、串通涨价等不法行为。

　　(二)推动服务业加快发展。积极推进国家服务业综合改革试点，加快构建信息服务、商贸物流、金融服务、文化旅游、商务会展五大区域性服务业中心，服务业增加值占生产总值的比重提高1个百分点以上。一是抓投入。千方百计扩大服务业投资，完善服务业发展激励机制，用好专项引导资金，强化财税金融支持，引导更多社会资本投向服务领域，集中抓好一批技术含量高、市场前景好的服务业大项目。二是抓优势产业。信息服务业以“数字泉城”、中国软件名城建设为抓手，加强信息基础设施建设，加快推进三网融合，大力发展软件服务外包，打造浪潮、中创和国家动漫产业基地等一批信息服务业重点企业和园区。商贸物流业围绕打造全国性商贸物流中心城市，科学系统规划传统商贸流通业布局，推进一批重点物流园区建设，扶持重点商贸企业加快发展。金融服务业以促进区域金融中心建设为核心，积极引进金融机构，加快发展新型金融机构，大力发展金融要素市场，构建良好的金融生态环境，防范金融风险。文化旅游业以培育特色、创建品牌为重点，完善设施，整合资源，加快发展现代传媒、文化旅游等七大文化产业，推动30个文化产业重点园(街)区建设，打造“天下泉城”旅游品牌。商务会展业坚持专业化、社会化和市场化方向，加快发展财务、法律、咨询和产权交易等新兴服务业。商贸餐饮业、农村服务业、社区服务业要落实好有关政策意见，突出发展重点，提高规模水平。三是抓载体。市中、历下、天桥、槐荫、长清、历城、章丘等7个重点城区在扩张规模、优化结构的基础上率先发展，服务业比重提高2个百分点以上，其他县区提高1个百分点以上。增强齐鲁软件园、泉城路商业集聚区等30个重点园区的集聚力和带动力，扶持100家重点企业做大做强。优化服务业发展环境，完善统计监测，健全考核体系。

　　(三)促进工业优化升级。坚定不移地把工业作为转方式调结构的重点，推进主导产业高端化、新兴产业规模化、传统产业升级优化，加快淘汰落后产能，提升核心竞争力。一是培育战略性新兴产业。启动综合性国家高技术产业基地核心区规划建设，打造“一城三区四基地”空间发展格局。围绕加快新信息、新能源及节能环保、新医药及生物、高端装备制造四大新兴产业发展，加大政策和资金扶持，加快项目培育和引进，集中突破一批制约产业发展的关键核心技术。发挥国家信息通信国际创新园、齐鲁软件园的优势，发展物联网、云计算和高端软件产业，抓好千万亿次超级计算机、量子通信技术、卫星通信项目。扶持以光热光伏、风电装备、垃圾发电综合利用为重点的太阳能、风能、生物质能项目开发利用，发展以生物疫苗、作物育种、酶制剂为重点的生物医药、生物农业、生物制造产业，提升以数控机床、压力机等产品为重点的高端装备制造业。二是加快传统产业改造提升。突出抓好冶金、石化、食品、建材、轻工等产业技术改造，做优做强重卡、轿车、摩托车和高端柴油机等机械装备产业，加快技术、产品、经营管理和营销模式创新。实施重点技术改造项目计划，加快174个调整振兴重点项目建设，确保技术改造投资占全市工业投资70%以上。三是加强园区载体建设。继续支持高新区加快发展，努力打造全国一流的自主创新核心区、高层次人才集聚区、战略性新兴产业和先进制造业发展示范区。增强8个省级开发区承载带动能力，强化项目引进，提升产业层次。四是提升自主创新能力。深入开展关键技术和共性技术研究，加强科技服务体系和科技资源共享平台建设，实施重大科技专项和专利、品牌、商标、标准战略，培育一批重点品牌企业和中国驰名商标。全市各级企业技术中心、工程技术研究中心达400家以上，培育150家创新型企业和500家成长型科技企业。抓好企业家队伍建设。

　　(四)统筹新农村建设工作。坚持以工促农、以城带乡，启动统筹城乡发展七大工程。一是大力发展都市型、城郊型现代农业。实施6亿斤粮食增产规划和蔬菜、畜牧、种苗、林果花卉四大产业振兴规划。打造绕城都市农业圈、南部山区生态观光农业带、中部平原特色精品农业带、黄北设施高效农业带，建设提升60个都市农业园区和40个特色品牌基地。抓好“菜篮子”工程，健全重大动物疫病防控、标准化生产、市场准入等6大体系，打造“农产品质量安全放心城市”品牌。二是拓展农民增收渠道。发展农产品加工流通业和农村家庭工副业，提高非农收入比重。强化农村劳动力技能培训，加快农民转移就业，增加工资性收入。落实好强农惠农政策，深化政策性农业保险试点，增加转移性收入。推进产业扶贫开发，实现6万贫困人口人均增收1100元。三是提升农村基础设施承载力。把农田水利作为农村基础设施建设的重点，大幅增加水利投入，突出抓好水毁工程修复及重点中小河道治理、山洪地质灾害防治、易灾地区生态环境综合治理等防洪薄弱环节建设。实施绣江河、土马河、大寺河、北大沙河等7条中小河流治理，抓好8000公顷易灾地区生态环境综合治理，解决15万人饮水安全问题。推进国家森林城市创建，重点抓好荒山绿化、退耕还林、平原风沙治理、水系生态绿化工程，完成造林13000公顷。加强农业气象服务、农村防灾减灾工作和黄河堤防建设。四是推进农村发展体制改革。加快乡(镇)农村土地流转服务中心建设，扶持15家市级示范中心，发展多种形式的适度规模经营。五是发展壮大县域经济。推动市内工业向县域转移，落实好继续支持商河加快发展的15条措施，加大远郊县与中心区对接力度，加快实施县域村镇体系规划，统筹安排农村旧村改造工作，抓好中心城镇建设。六是全力抗旱夺丰收。针对去冬今春特大旱情，把抗旱夺丰收作为当前最重要、最紧迫的任务，动员群众和社会力量，强化保障措施，加大资金投入，搞好抗旱工程建设，保障抗旱物资供应，合理调度水资源，科学组织春季农田管理，确保人畜饮水安全和农业生产顺利进行。

　　(五)提升城市建设水平。更加注重城市载体功能和产业统筹发展，更加注重突出城市特色、文化内涵和生态绿化建设，塑造现代化省会城市新形象。一是优化提升老城区。以释放发展空间、提升功能形象为重点，抓好棚户区、城中村和危旧简易楼群改造，积极稳妥地推进明府城、商埠区等重点区域的保护改造。二是加快建设三大新区。东部新区加快奥体文博、贤文、汉峪、唐冶等片区开发建设，推进服务外包基地、孙村高端产业区建设，完善市政配套设施，强化政务服务、体育休闲、商务办公功能。西部新区以京沪高铁济南西客站建成使用为契机，完成综合客运枢纽工程建设，加快核心区、济西湿地生态区、大学科技园、经济开发区等片区开发。滨河新区全面完成小清河综合治理二期工程，启动泺口、北湖、华山、新东站等片区规划建设。加快推进16个城市综合体建设，打造一批代表省会形象和品位的地标性建筑。三是强化城市基础设施建设。积极推动省会城市群城际轨道交通规划建设，规划建设长清黄河大桥、济齐黄河大桥、泺口穿黄隧道、石济客专黄河公铁大桥等重大基础设施，开工建设济商、青兰等高速公路，完成国道220线平阴段改建。完善中心城区路网结构，加快市内快速公交规划，实施二环西路高架、张庄路、刘长山路等20条道路建设改造。优化支路微循环体系，优先发展公共交通，构筑快速路网、智能交通系统，突出解决好交通拥堵问题。加强市政公用设施建设，实施济西二期供水工程和东区水厂建设，加大老城区供水、供电、供气、供热设施改造建设。完善城市安全保障体系，健全城市排水防洪系统，加大对防火、防震、气象等设施的投入，加强管沟等地下空间管理及集约化利用。四是加强城市景观建设和环境综合整治。抓好名泉保护利用，加快实施南部生态功能区保护工作，规划并统筹解决好城市生产、生活用水问题。实施市容环境专项整治，抓好拆除违章、户外广告、乱倒渣土、城乡环境、铁路沿线等综合整治，解决占道经营、开放式小区管理等热点问题，加快污水和垃圾处理厂、停车场等公共设施建设，推进城乡环卫一体化建设，提升环卫保洁、市政管护、集贸市场管理水平。

　　(六)推进节能减排和环境保护。树立绿色、低碳发展理念，加快构建资源节约、环境友好的生产方式和消费模式。一是狠抓节能降耗。强化源头控制，突出抓好钢铁、水泥、电力、化工等重点行业以及建筑、交通、公共机构等重点领域的节能降耗，大力推广节能技术、产品和装备，支持激励低碳技术研究应用，继续做好淘汰落后产能的各项工作。二是强化环境保护。以生态城市建设为抓手，严格执行环境准入和排污许可制度，倡导绿色消费和低碳生活方式。深入推进扬尘、机动车、建筑工地、小烟囱污染治理，实施重点企业脱硫设施再提高工程，突出抓好城区大气污染防治。加强水系生态建设，加大饮用水源地专项整治力度，加快十六里河、南大槐树沟等市区8条重点河道截污整治，确保小清河水质指标达到省里下达的化学需氧量和氨氮排放标准。严查各类环境违法行为，强化对57家市控污染源重点监管，实现省控重点企业排放达标率98%以上，确保完成主要污染物总量减排年度目标任务。统筹城乡污染防治，加快县城、乡镇污水处理设施建设，控制农村面源污染，提高农作物秸秆综合利用水平。三是强化资源节约和综合利用。大力发展循环经济，积极推行绿色生产和清洁生产，在重点行业、领域和产业园区开展循环经济试点，搞好工业、建筑、城市生活垃圾等废弃资源综合利用，力争工业固体废物综合利用率达到95%以上。积极推进分质供水工程，加大中水回用、污水再生利用和雨洪水利用。四是强化责任考核。严格落实节能减排目标管理责任制，明确各县(市)区、各部门和重点企业的责任，加强指导，强化督查，确保不折不扣地完成省里下达的节能减排任务。

　　(七)切实保障改善民生。今年政府财政支出新增部分重点用于保障改善民生。一是千方百计促进就业。实施就业优先发展战略，多渠道开发就业岗位，加大技能教育培训力度，积极推动以创业带动就业，重点做好高校毕业生、农村转移劳动力、城镇就业困难人员就业工作。建立健全统一规范的人力资源市场，完善城乡平等就业长效机制。新增城镇就业10万人、农村劳动力转移就业5.2万人、援助就业困难人员1.2万人。二是完善社会保障体系。进一步扩大社会保险覆盖面，市级财政投入2.2亿元，实现新型农村社会养老保险全市280万适龄人员全覆盖。市级财政投入2.1亿元，把新农合政府补助标准提高到每人每年200元。全市人均基本公共卫生服务经费标准由15元提高到25元。完善城镇居民养老保险制度，进一步提高医疗、失业、工伤和生育保险统筹层次，城镇基本医疗保险实现市级统筹。提高企业退休人员养老金水平，月人均普遍增加160元。提高失业保险金标准，扩大工伤保险适用和认定范围，调整工伤人员伤残津贴、护理费和供养亲属抚恤金标准。建立城乡低保和农村五保供养标准自然增长机制，实现我市城乡低保、五保供养标准与经济社会发展水平同步增长。保证集中和分散抚养孤儿每月基本生活费分别不低于1000元和600元。继续提高企业最低工资标准，两个档次分别提高到1100元和950元，比去年平均增长16.4%。健全社会救助和保障标准与物价上涨挂钩的联动机制，落实好优抚对象、低保对象、农村五保对象、家庭困难学生临时价格补贴政策，保证困难群体基本生活需要。扶持慈善事业发展，健全教育、医疗等专项救助制度，加快发展社会养老服务，大力培育以社区为依托的居家养老服务模式，建设市级养老服务中心。加快建立残疾人社会保障和服务体系，保障妇女儿童合法权益。加强计划生育服务工作，深入开展计生“基层基础建设落实年”活动，抓好全国流动人口基本公共服务均等化试点，稳定低生育水平。三是推进住房保障体系建设。调整保障性住房供给结构，全年保障性住房建设达到3万套以上，其中新建公共租赁住房2.3万套以上，提供2000套以上廉租住房，全面完成农房建设改造任务。四是制定实施《济南市中长期教育改革与发展规划纲要》。切实办好学前教育，实施幼儿园标准化建设工程，新建50所公办幼儿园。继续优化中小学布局，推进中小学校舍安全工程，规范城区新建居住区教育设施配套建设。重视提高中小学生身体素质，切实减轻课业负担。完善高中和职业教育，加快技师学院建设，完善提升章丘高等职业教育基地，扩大中等职业教育免费范围，加大农村教育投入，积极争创国家级重点职业学校和国家级示范中等职业学校。进一步提高全市大中专、中小学生均拨款水平，将农村初中和小学的生均公用经费补助标准、寄宿生生活补助标准分别提高100元和250元。五是加快发展文化体育事业。以筹办第十届中国艺术节为契机，加快省会文化艺术中心等重大文化设施建设，今后三年投资5600万元进一步完善基层文化服务设施。继续实施“文化精品工程”，开展文化惠民八大主题行动，培育健康的网络文化。大力开展全民健身活动，逐步推动文化体育设施免费开放。在前三年为民办实事基础上，今年继续扎实办好10件实事。

　　(八)深化改革扩大开放。推进重点领域改革取得新突破。一是继续深化国有企业改革。推动市属国有资产战略重组，逐步实现市属经营性国有资产集中统一监管，健全国有资本经营预算制度。加强对非公有制企业的服务和扶持。二是加快医药卫生体制改革。深入开展公立医疗机构改革，扩大基本药物制度实施范围，研究制定乡村医生补偿机制，推进卫生强基工程，提高传染病预防和救治能力，提高中医药服务能力。三是加快文化体制改革。推进经营性文化事业单位转企改制和整合重组，创新公共文化服务运行机制。四是推进要素市场改革。健全公用事业价格形成机制，稳步实施资源性产品价格和环保收费改革。引导行业协会中介服务社会化、经营运作市场化。加强投融资平台改革与管理。深化机关后勤服务社会化改革。

　　进一步提高对外开放水平。一是培育对外贸易发展优势。继续稳定和拓展外需，推进加工贸易转型升级，优化对外贸易结构，提高自主知识产权和高技术含量、高附加值、高效益商品出口比重，扶持出口重点行业、重点市场、重点商品和重点企业。优化进口结构，扩大进口规模。大力发展服务贸易，加快发展服务外包产业。二是提高招商引资质量。积极承接国际国内产业转移，提高利用外资水平，引进国际知名企业来济设立地区总部、制造基地、研发中心、采购中心和服务外包基地，大力引进和培育海内外高层次、高技能人才。三是扩大对外交流合作。加快“走出去”步伐，引导企业有序发展境外投资、工程承包和外派劳务。深化友城交流，促进城市间合作发展。四是加强口岸建设。加快济南国际机场指廊建设，增加国际航班密度，筹建济南铁路口岸，推进济南综合保税区建设，实施大通关工程。

　　(九)维护社会和谐稳定。创新社会管理机制，营造平安稳定的社会环境，服务和保障省会建设发展。一是推进平安济南建设。启动“六五”普法规划，深化社会治安秩序专项整治，加快动态立体防控体系建设。健全社会稳定风险评估机制，完善领导干部定期接访、下访制度，深入开展矛盾纠纷排查化解。加强公正廉洁执法和执法规范化建设，扩大法律援助覆盖面，保障弱势群体合法权益。二是强化基层基础建设。加强村、居和社区建设，健全服务设施，完善便民网络，探索管理新模式。三是高度重视公共安全。加大投入，健全应急体系，做好地震和极端天气等自然灾害预警防范，强化地下管网、公共避灾场所和人防工程建设管理。加强安全生产监管，深化矿山、道路交通、建筑施工、危险化学品、烟花爆竹、人员密集场所消防安全等专项整治，推进安全社区、安全校园建设。强化公共卫生和食品药品安全监管，做好疫情防控工作。四是加强精神文明建设。继续开展“爱泉城、讲文明、树新风、创品牌”六项文明工程，做好社会志愿服务工作，促进精神文明创建常态化、科学化、制度化。加强民族团结和宗教工作，完善双拥优抚安置服务保障体系，努力推动全国双拥模范城建设。

　　(十)加强服务型政府建设。全面完成今年和“十二五”规划目标任务，必须进一步改革创新，努力建设人民满意的服务型政府。一是深入转变政府职能。强化社会管理和公共服务，深化行政审批制度改革，完善网上审批和电子监察系统，简化程序，提高效率，优化环境。强化行政问责和效能监察，健全绩效评价考核体系，提高政府公信力和执行力。二是坚持依法科学民主决策。全面推进依法行政，自觉接受市人大及其常委会监督和政协民主监督。健全群众参与、专家咨询和政府决策相结合的决策机制，加大决策跟踪反馈和责任追究。加强网上政府和市民服务热线建设，完善信息公开和新闻发布制度。三是深入推进作风转变。牢固树立群众观点，畅通社情民意反映渠道，建立健全问政于民、问需于民、问计于民的长效机制。大力精简会议和文件，弘扬求真务实的作风，深入基层、深入实际，察实情、出实招、办实事、求实效，把为民造福的工作紧紧抓在手上，把促进发展的任务坚决落到实处。四是加强政府反腐倡廉建设。全面落实党风廉政建设责任制，强化惩治和预防腐败体系建设。加强对各级公务员队伍的教育和监督，提高拒腐防变的能力。严格落实领导干部廉洁自律各项规定，加大查办违纪违法案件工作力度，严肃查处腐败分子。加强对中央和省、市重大决策部署执行情况的监督检查，强化政府投资项目的审计监督和行政监察，严格领导干部经济责任审计。推进专项治理工作，继续深化工程建设领域突出问题和“小金库”专项治理，开展庆典、研讨会、论坛过多过滥问题和公务用车问题专项治理。推进廉政文化建设，营造风清气正、干事创业的良好氛围。

　　各位代表！未来五年是我市深入推进科学发展、全面建设小康社会的重要战略机遇期。让我们更加紧密地团结在以胡锦涛同志为总书记的党中央周围，高举中国特色社会主义伟大旗帜，深入贯彻落实科学发展观，在中共济南市委的坚强领导下，解放思想，提升境界，开拓进取，真抓实干，为全面实现济南市国民经济和社会发展第十二个五年规划和全面建设小康社会宏伟目标而努力奋斗！
